# A qualitative study of physician perspectives on adaptation to electronic health records

**DOI:** 10.1186/s12911-020-1030-6

**Published:** 2020-02-10

**Authors:** Cynthia J. Sieck, Nicole Pearl, Tiffani J. Bright, Po-Yin Yen

**Affiliations:** 1 Department of Family Medicine, The Ohio State University College of Medicine, Columbus, OH 43201 USA; 2 The Center for the Advancement of Team Science, Analytics, and Systems Thinking, Columbus, OH USA; 3 Institute for Informatics, Washington University School of Medicine, St. Louis, MO USA; 4 IBM Watson Health, Cambridge, USA; 5 Goldfarb School of Nursing, Barnes-Jewish College, St. Louis, MO USA

**Keywords:** Health information technology, Adaptation, Electronic health records

## Abstract

**Background:**

Electronic Health Records (EHRs) have the potential to improve many aspects of care and their use has increased in the last decade. Because of this, acceptance and adoption of EHRs is less of a concern than adaptation to use. To understand this issue more deeply, we conducted a qualitative study of physician perspectives on EHR use to identify factors that facilitate adaptation.

**Methods:**

We conducted semi-structured interviews with 9 physicians across a range of inpatient disciplines at a large Academic Medical Center. Interviews were conducted by phone, lasting approximately 30 min, and were transcribed verbatim for analysis. We utilized inductive and deductive methods in our analysis.

**Results:**

We identified 4 major themes related to EHR adaptation: impact of EHR changes on physicians, how physicians managed these changes, factors that facilitated adaptation to using the EHR and adapting to using the EHR in the patient encounter. Within these themes, physicians felt that a positive mindset toward change, providing upgrade training that was tailored to their role, and the opportunity to learn from colleagues were important facilitators of adaptation.

**Conclusions:**

As EHR use moves beyond implementation, physicians continue to be required to adapt to the technology and to its frequent changes. Our study provides actionable findings that allow healthcare systems to focus on factors that facilitate the adaptation process for physicians.

## Background

Electronic health records (EHRs) have the potential to decrease medical errors, improve communication among healthcare providers, and improve coordination of care [1–3]. Since first introduced in 2009, implementation of electronic health records has increased steadily over the last decade due to policies to encourage use [4]. The US has spent over $20 billion in incentives to implement and use EHRs [1]. Policy efforts such as the Health Information Technology for Economic and Clinical Health Act (HITECH) and measures of Meaningful Use of technology have pushed the implementation of EHRs across all healthcare domains [5, 6].

However, physicians still face barriers to use including interfaces that are not user-friendly, lack of interoperability between healthcare systems, changes to workflows required for implementation and a perception by physicians that EHR use does not always equate with improvements in quality [4]. In fact, a recent survey shows that only 20% of physicians felt meeting Meaningful Use standards would improve care [7]. Physician express frustration about the impact on face-to-face interactions with patients and acknowledge decreased fulfillment in their jobs [2, 8, 9]. However, in other studies, physicians also note positive impacts including easier access to information, particularly remotely, and better quality of care, suggesting mixed opinions about the impact of EHRs on qualityof care [10]. The process of acknowledging the benefits and challenges of EHRs and adapting to their use has been equated to a progressing through the stages of grief model [11] in which initial resistance is eventually overcome resulting in acceptance.

Further, implementation of the EHR is not limited to a single event in time. Rather, the technology changes when newer features are added or interfaces are redesigned. These upgrades may improve functionality, but they also require physicians to adapt to changes beyond the initial implementation. Users are required to continually learn how to use the newer system and then to incorporate these upgrades into their clinical workflow, often with negative work and psychological impacts. Studies suggest that this type of continual change, such as that required by changes to the EHR, can result decreased productivity, increased stress and increased burnout [12–14].

Implementing EHRs requires understanding the culture and context in which use will occur, not just the technical elements of implementation. Studies of EHR implementation focus on successful adoption but do not adequately capture what happens after physicians begin using the EHR, or how physicians adapt to use. Defined as “a process of modifying existing conditions in an effort to achieve alignment,” adaptation reflects the development, installation and maintenance of an innovation as well as the new procedures and training required to support use of an innovation [15, 16]. This is distinct from the optimization phase of technology implementation as it refers to the processes individual users employ to incorporate the technology into their own workflows, compared to optimization which examines strategies at an organizational level [17]. Adaptation follows implementation and includes training in using the new technology as well as workflow procedures surrounding its use. In Donabedian’s quality assessment model examining Structure-Process-Outcome related to quality improvement, adaptation occurs in the Process step in which clinician use of the EHR is examined [18]. Within this process step, we can identify factors such as institutional training provided, acceptance of technology, communication and collaboration among clinicians, impacts on work productivity, health system policies related to EHR use and leadership [16]. Thus, considerations of the culture and context in which adaptation occurs is critical to understanding the process.

Adaptation is not well understood in the context of EHR implementation. Much EHR-focused research examines acceptance and adoption of technology. For example, studies often examine technology use through the Technology Acceptance Model (TAM) which considers perceived usefulness, perceived ease of use, attitude toward using and behavioral intention to use a particular technology as predicting actual technology use [19, 20]. The Extended TAM (TAM2) includes factors such as output quality, social environment, experience of use and voluntariness of use of technology to understand intention to use and actual use [21]. While studies demonstrate a relationship between TAM and TAM2 constructs and use, these models do not adequately describe what happens as one begins to use the technology and how end users then adapt to its use [22]. This is particularly relevant to EHRs because upgrades to the system occur regularly and can have a significant impact on physician workflow and their experiences.

### Objectives

To improve our understanding of this next phase of use, adaptation to EHR technology, we conducted a qualitative study of physicians’ perspectives related to adaptation to use of an EHR to identify factors that facilitate adaptation during initial implementation as well as adaptation required when the EHR system is upgraded. Our goal was to obtain a range of perspectives across specialties to identify common adaptation factors rather than to compare factors relevant to one specialty to another.

## Methods

### Sample and setting

We conducted semi-structured interviews with hospital-based physicians at a large Academic Medical Center (AMC) in the Midwest. The AMC implemented the Epic Systems EHR (Verona, WI) system-wide in 2011. The EHR is in use in all inpatient and outpatient practices. The AMC operates on a regular upgrade schedule in which minor upgrades may take place quarterly with larger upgrades bundled into an annual upgrade and provides notification to all care team members through multiple venues including email, login screen notification and discussion at departmental meetings.

We focus on physicians from any specialty practicing in the inpatient setting because use of the EHR may be different in the inpatient and outpatient setting, with the inpatient setting requiring potentially different interaction with the EHR than may be necessary in an outpatient setting. In addition, the roll out process used for inpatient and outpatient settings was different which may have influenced adaptation. Because workflow and EHR use patterns may vary by specialty, we sought to include a range of physician specialties in our sample to obtain as broad of a view as possible. Interviewees were identified by a snowball sampling method in which each interviewee was asked to identify colleagues who might be willing to discuss their perspectives on incorporating the EHR into their practice. The research team met regularly throughout the data collection process to determine when saturation of concepts was reached.

### Data collection

The semi-structured interview guide included questions focused on 5 main topics: physician background information including area of specialty, years of use of EHR and self-described comfort with EHR; frequency, format and perceptions related to training in using the EHR; impact of EHRs on communication with departmental colleagues, colleagues across the AMC and outside of the AMC; impact on work productivity; and perspectives about technology-related policies. Interviews were conducted by telephone and lasted approximately 30 min. All interviews were audiorecorded and transcribed verbatim.

### Analysis

We utilized both inductive and deductive methods using the constant comparative analytic approach [23]. Two members of the research team developed a preliminary coding dictionary based on the semi-structured interview guide and applied this dictionary to double code five interviews, reconciled any coding differences, then coded the remaining interviews independently. Coders met to discuss each coded transcript and compared themes, expanding the coding dictionary as new themes were identified. The entire study team met regularly to discuss coding progress, identify and resolve discrepancies and reach consensus. We used Atlas.ti (version 6.0) qualitative data analysis software to support our analysis.

## Results

### Description of interviewees

Table 1 provides a demographic description of interview participants. Interviewees represented a range of physician specialties, had been practicing medicine for between 6 and 28 years, and were slightly over 50% male. All except one participant reported being moderately or very comfortable with the EHR. Our analysis did not identify differences in the themes described below by specialty, years of practice or comfort with the EHR.

**Table 1:** Participant demographics

Specialty	Sex	Years at AMC	Comfort with EHR
Infectious Disease	Female	13	Very
Hospitalist	Male	8	Very
General Surgery	Female	6	Moderately
Hospitalist	Male	9	Moderately
Emergency Medicine	Female	6	Moderately
Gastroenterology	Male	26	Moderately
Neurology	Female	26	Moderately
Pulmonology	Male	28	Very
Dermatology	Male	8	Moderately

We found that physicians noted specific benefits to using an EHR. For example, one physician noted the ease of using the EHR while conducting rounds, “*As it relates to patient care, it makes it much easier. I was actually on the floor rounding when you called initially. It makes it easier. Certainly we can pull up any data any time that we need it. We have those little computers on the walls and stuff. It’s not like we have to go back to the main nursing desk to get the information*.” Another noted that communication with patients and outside the health system can happen significantly faster, “*It probably gets there faster. In the old days, I used to dictate a letter. In the 1990s I had a Dictaphone. I would see a patient, I’d dictate a letter, and then it would be transcribed. Then I had to sign it, and it was sent out. That might take a week*.” Finally, another noted the increase in efficiency of communication among colleagues, “*I think it gets a—the information a little bit more at your fingertips and embeds it in a place you’re already looking for the information, anyhow. I think it’s truncated the notes, so we’re not getting as verbose in the notes. I think that’s been one benefit where some of the communication between partners to be able to flag those kinds of things*.”

In addition, we identified four major themes related to managing change from EHR implementation: impact of changes to the EHR, dealing with changes, factors that facilitate adaptation, and impact on patient care. Below we describe subthemes identified in each of these major themes with example quotations.

#### Theme 1. Impact of changes

We also examined how use of an EHR impacts physicians and ways in which that requires physicians to adapt their practice. Some felt the impact was minor while others reported a more substantial impact. For example, one physician told us,” *Then the other thing that’s very frustrating people, once you get in a groove, let’s say you know what you’re doing, it’s working for you, and then there’s these major up changes that they just change everything from what the screen looks like and so forth… Not that it’s never gonna change, it will, but just to make it so crippling to you, is just—people complain about it*.” Another acknowledged that changes cause an initial disruption but they could quickly adjust, “*I would say upgrades in the electronic health record are more impactful. Because those happen pretty often, and sometimes they’re subtle. But, they’re enough to slow you down for a couple days. Even changes in color and changes in where the buttons are will—it’s surprisingly disruptive. You get right back up to where you were, as long as it’s the same system that started out with. So, it’s not like it’s a long-term disruption, but those are the things that I think are more day-to-day disrupting*.”

#### Theme 2. Dealing with changes

##### Communication received about change

We also examined how changes to the EHR were experienced by physicians. A common theme related to how changes were communicated to physicians. As one physician told us, “*What I think would be really helpful is sort of periodic updates. If there’s going to be a massive change or update, or their templates are changing, approaches are changing in the EHR, if they could come to our division meeting, department meetings, probably division meeting, or even our clinics, just to give us a, ‘Hey, heads up. This is how you navigate this. A, B, or C.’*” Others felt that email communication about changes was so frequent that it lost its effectiveness. For example, one physician said, “*I hate to say it that way, because we get, there’s email fatigue, too. I’m not going to lie. Just like there’s alarm fatigue, there’s email fatigue. A lot of times I don’t even open ‘em. I delete a lot of stuff. You just gotta’ focus on stuff that seems relevant for the moment to get you through the day*.”

To manage email fatigue, some physicians noted that they prioritize which messages to read, often only paying attention to final warning emails. As one physician told us*“…what’s good about the place is they definitely do give us multiple emails for things that are super important. They know we probably delete a lot. Then you get the final warning, big capital letters, exclamation marks. Those are the ones I usually open up just to figure out what’s going on.*”

#### Theme 3. Factors that facilitate adaptation

Within the theme of factors that facilitate adaptation, physicians discussed maintaining a positive mindset toward change, seeing benefits of the EHR, tailored training and physician voice in modifications, and learning from colleagues as facilitating adaptation to the EHR. Table 2 below provides example quotes and potential improvement strategies associated with each factor.

**Table 2:** Specialty, Sex, Years at AMC and Self-reported Comfort with EHR

Adaptation factors	Example quotes	Potential improvement strategies
Positive Mindset toward Change	*“Understanding how to do your job is part of being a doctor, and part of being a good doctor.”*	• Cultivate specialty-specific physician champions
	*“The last big change that I remember was when they changed all the templates, the font, and everything looked different. It didn’t really impact it too much. Maybe the first two patients that I did in clinic. It only really affects me in clinic because that’s where I’m doing all my major notes.”*	
Recognizing Benefits of the EHR	*“I mean despite all the stuff I just said, nobody is gonna recommend that we go back to paper. I think everybody understands the value of it, and this is the direction. It is a good thing. I can see all the things.”*	• Highlight benefits of EHR and upgrades in all communication efforts • Provide explanation for why changes are made
	*“Yeah, it’s better. It’s not paper charts. Again, I remember paper charts. Everybody complains about, oh, EHR takes so long. No. Paper charts take forever’”*	
	*“… we often didn’t do it [chart temperatures], because it was just too burdensome to do on a daily basis. So, we’d just do it occasionally when we’re trying to figure it out. But, now we have this extra information available to us at all times. I’m not sure if I know if that’s helpful to patients or not. It seems helpful.”*	
	*“I think from an efficiency standpoint, I can see, definitely, gains there.”*	
	*“Prior to that, we were using—to my knowledge, at least—three different, coexisting systems. … it was highly inefficient.”*	
	*“I mean to put that into perspective of what it was like in the 1990s, every service had their own chart. I had a chart from a patient I saw in gastroenterology, and the patient was also seeing a cardiologist and a rheumatologist. They had their charts. There was no unified chart.”*	
Tailored training and physician voice in modifications	*“At least in the Department of Emergency Medicine, we’ve managed to get several people onto different committees.”*	• Provide general as well as specialty-specific training • Incorporate stakeholder input into training development
	*“If it was actually specific for my specific inpatient job, like let’s just talk about consultants, and we had some say in it.”*	
	*“I think that they feel like if it’s somebody from our division, they have our best interests in mind. Not to say that somebody outside the division couldn’t do that training, but I think it would have to be—it would likely need to be connected with somebody in the division, so it feels like it’s more personalized.”*	
	*“I think it works well for us because it’s someone that we know that inherently knows our workflow. Sell that idea and efficiency to our group.”*	
	*“It has all these stock phrases that make life so much simpler… They made ‘em, and then I stole them.”*	
Learning from Colleagues	*“It’s usually things like at a division meeting someone will say, I can’t figure out how to do this, and someone else would say, oh, you just have to do this and this and this.”*	• Create electronic mechanisms that facilitate sharing of stock phrases • Allow time for colleagues to share challenges and solutions
	*“I just had some other colleagues show me tips or tricks, and you can use the share function to steal tools other people have made.”*	
	*“I think you need some initial basic instruction, but you learn more on the job from your colleagues.”*	

##### Subtheme 3.1 positive mindset toward change

Physicians recognized that EHRs are now a necessary part of care that will remain and many felt that simply accepting this and learning to use it well helped them to adapt. As one physician told us,” *Yeah, I mean to be honest with you, mostly it’s a mindset. You just come. If I’m gonna do this, I’m gonna have to learn to do it.*” Another discussed the importance of a positive attitude this way, “*Oh, you just have to do it. You just have to use it*.”

##### Subtheme 3.2 recognizing the benefits of the EHR

Related to a positive mindset about using EHRs, physicians noted that recognizing the positive elements of using EHRs encouraged them adapt to use. For example, one physician noted, “*I don’t have to look at the paper chart. I still remember when paper charts were—so, this is a thousand—as bad as EHR can be, it’s a thousand times better than paper charts.*”

##### Subtheme 3.3 tailored training and physician voice in modifications

Physicians noted that training tailored to their role helped them to adapt to using the EHR. For example, as one physician told us, “*I think if it’s very specific, and it’s sort of triggered by the provider… then they think, they’d probably accept it pretty well. But, if it’s sort of imposed, probably not*.” Another described a helpful training interaction this way, “*They had representatives from nursing, from our nursing assistants, physicians, and leadership, and said, “This is our thought based on what was done on other units. How would you like to adapt this to your unit?* “*That seemed to go much better*.” Physicians from different specialties also described the benefits of having a representative from their department participating on EHR-focused committees to ensure their specialty’s perspective was represented when changes or upgrades are planned. One physician told us, “*At least in the Department of Emergency Medicine, we’ve managed to get several people onto different committees..”*

##### Subtheme 3.4 learning from colleagues

The final adaptation strategy we noted was the opportunity to learn from colleagues through sharing of tools or observation. Because the initial training provided is often generic in nature, physicians noted they had to learn how to tailor use of the EHR to their own style, which was facilitated by learning from colleagues. For example, one physician told us,” *I went and watched people in clinic. People who were already doing it. I also sat down with some other physicians, and they gave me little tips on what they did to make it better*.”

#### Theme 4. Impact on patient care

Physicians discussed ways in which the availability of the EHR influenced patient encounters. Some physicians described ways in which the EHR made inpatient care more efficient. For example, one told us, “*And back when I used paper charts, for better or worse, you really only had the talking to the patient, trying to get their information, maybe talking to one of their physicians and then reading through the chart, which even my huge, huge chart was a limited amount of work.”* Physicians also felt the EHR made patient care safer, as one stated, “*I think it has probably improved safety of patients, because we have—we actually know more, you’re not guessing. You’re not taking people’s words for it. You actually have the person’s records from five years ago, for example. You actually know what happened.”*

Several physicians noted that while the EHR made information more readily accessible, this information also introduced new challenges. One physician described balancing the volume of information available electronically with gathering information from the patient in this way, “*But, I mean, it’s not uncommon to think that you have an entire picture of what’s been going on, when you’ve look at the chart in-depth, and actually then get up and go and see the patient, find out that that’s not at all what’s going on.*”

Some physicians, however, felt that using an EHR resulted in decreased patient contact. For example, one physician told us, “*You can get such a good history from a chart, the patient was just in the hospital, and so you know day-to-day what happened, and you’re writing up this whole timeline, and you know everything that happened. Your actual evaluation of the patient might be a little shorter. You might just sort of say, okay, so, I understand from your chart that this, this, this, happened. Is that true? And they say, sure. And then you ask them a few more questions. As opposed to really talking to them for a long period of time*.” Another told us, “*Well, you just can’t see as many patients. You can’t get as many in… You’re documenting in the computer. You’re doctoring the computer*.”

To manage the impact on patient care, physicians utilized different approached depending on the situation. When patients became frustrated at being asked to repeat information they felt should be in the EHR, one physician would tell patients *“…there’s something to be gained from you telling me, because one, I can figure out whether or not that’s—you understand what’s going on, to some extent, and maybe you went somewhere else and I don’t know about that.*” In response to the need to face the computer while asking the patient question, one physician stated, “*I make jokes. I say, “How is the back of my head looking?” What am I supposed to do? I apologize. Sometimes I’ll move the patient. Instead of having them—I say hey, why don’t—especially if it’s somebody who I don’t know, or if it’s gonna be a lengthy conversation, I’ll say why don’t you sit over here, and I’ll move a chair so it’s to the side of me, so that I can periodically look at the computer and look at them.*”

## Discussion

Our study extends beyond initial adoption and implementation of EHRs to examine ways in which physicians adapt to using this tool in new workflows. Physicians in our study noted benefits of using EHR, which are supported in existing literature. They felt the EHR increased efficiency and speed, as well as provided an accessible source of information. These benefits have also been noted in other studies [3, 24–26]. While interviewees recognized these benefits, they also discussed ways in which it changed their practice and identified factors that helped them to adapt.

### Positive attitude

The impact of attitude on an individual’s response to change can be significant. A positive, accepting attitude toward change has been shown to result in engagement with changes required and more positive work behaviors [27, 28]. In our study, physicians identified that accepting the necessity of change in using EHRs positively influenced their ability to adapt to their use. Many noted that while they experienced frustrations with EHRs, they also recognized the benefits and thus felt more positive toward use. Other studies have identified strategies such as protecting time in the workday that does not involve interaction with the EHR and taking care of oneself outside of work as ways to maintain a perspective about adapting to EHR use [29, 30].

This finding also has implications for how EHRs and the accompanying upgrades are presented to physicians. Communication that helps physicians to see the benefits of EHRs and changes to the EHR could facilitate physician adaptation. For example, working with physician champions within a department who can model a positive attitude may help address specialty-specific concerns may facilitate adaptation among colleauges, a strategy that has been successful in facilitating interventions across various domains of health care [31, 32]. However, while physican champions may be effective for encouraging a positive attitude toward changes, they alone may not be sufficient. Organizations also need strong institutional leadership and a culture that supports clinicians in adapting to the changes associated with EHRs and associated upgrades [33, 34].

### Clinician involvement in EHR design

Incorporating stakeholders in the design process has gained acceptance across a range of disciplines [35]. Specific to EHRs, research shows that when clinicians are able to make significant contributions to the development of clinical content for EHR systems, they report higher levels of satisfaction when reviewing clinical content within their domain of knowledge [36]. Farley et al. suggest that EHR systems should be designed with clinicians involved at the front-line to ensure alignment with clinician perception and decision making [37], and no mechanism exists that allows or encourages end users to provide feedback about ongoing issues or concerns in the EHR. Furthermore, a growing body of literature exists that identifies poor EHR system design, rather than user error, as the more frequent cause of medical errors [37, 38]. In response to the lack of support in the traditional EHR development process, a non-profit organization created the open EHR approach [39]. Through this approach, clinicians are able to engage in the development and reviewing of standardized clinical content models, thus contributing to improved communication and quality patient care [36, 39]. Another study yielded similar findings using the open EHR approach, suggesting that successful EHR design is dependent on both technical and clinical competence, and clinicians and developers working together [40]. A user centered design approach could help realize the benefits of clinician involvement in EHR development and improve clinician satisfaction and quality of care [41, 42].

### Training tailored to clinician role

In addition to physician involvement in EHR design, including physicians in EHR training so that it can be tailored to their specialty can also help with adaptation. In the Technology Acceptance Model (TAM), training is an important concept because it provides an opportunity to influence the users’ perceived ease of use of the technology, thus increasing their acceptance of the technology [22]. In the context of EHRs, training that focuses on the specific applications to each specialty and workflow can improve perceived ease of use. In our study, physicians expressed a desire for training that focused on the particular ways physicians use the EHR, as compared to general training provided to all users, and reported seeking out representation on EHR-related committees in order to have their perspective included. In this way, training designed in collaboration with physicians to address physician-specific needs can increase perceived ease of use and help physicians adapt to using the EHR.

In our study, physicians noted concerns about communication about upgrades. While physicians expressed mixed views about how upgrades effect their productivity, with some expressing frustration and some accepting that upgrades are necessary and their productivity will recover, most wanted additional means of learning about upgrades and incorporating them into new workflows. Email and alerts at sign in were the most commonly mentioned means of communicating about upcoming changes to the EHR, but physicians frequently remarked that they did not find these means to be particularly effective. Because of the potential disruption caused by having to adapt to frequent and sometimes significant upgrades, healthcare systems need to find additional ways to support physicians in the process. The user centered design approach suggested above for development of the EHR can also be applied to communication related to EHR upgrades.

### Impact of EHR use on patient care

Finally, the impact of EHR use on patient care is still a concern. Physicians in our study noted that patient care was at the center of their desire to practice medicine and simply the presence of the EHR caused them to adapt how they manage these interactions. They noted descreased patient interaction and the need to adapt how they interacted with the patient. These concerns have also been noted elsewhere in the literature. For example, several studies have found adverse effects of EHR use on flow of conversation between the patient and physician. Margalit and colleagues found that physicians’ use of orientation statements (i.e., instructions and directions to the patient) decreased as their screen gazing and keyboard activity increased [43]. Similarly, additional studies discovered that entering data into the computer during the patient consultation was associated with lower levels of patient trust and patient satisfaction [44–46]. Other research has found that the style of physician-patient interaction shifted from a conversational style to a more “blocked” style due to data entry during the consultation, thus limiting the ability to build or maintain good rapport [47, 48]. These concerns are present not only during the initial EHR implementation but also resurface when upgrades change the way physicians interact with the EHR. The result is that physicians must not only adapt their workflow to incorporate the requirements of the EHR; they must also adapt their communication style in the visit to accommodate the presence of computer on which they use the EHR. When upgrades to the EHR change the user interface, the physician must become familiar with this while still engaging with the patient.

## Limitations

We note a few limitations to our study. First, our interviews were conducted at a single AMC and thus may not reflect the experiences of physicians at other healthcare systems. Secondly, while we interviewed physicians across a range of specialties, not all specialties were included in the interviews. It is possible that there are differences in adaptation by physician specialty that were not discovered in our study. Almost all interviewees were comfortable using an EHR. Given that length of time that EHRs have been in use, this is not unexpected. In addition, having achieved a level of comfort in using the EHR may enable users to better reflect on the process by which they adapted to ERH use. Finally, the snowball sampling method may reflect a bias in terms of who is suggested for inclusion into the study. We chose this method because it allowed participants to nominate colleagues who had expressed both positive and negative perspectives about EHR use.

## Conclusion

EHR use continues to increase across the US and the technology undergoes frequent upgrades. Thus, physicians continue to be required to adapt to the technology and to its frequent changes. We found that physicians felt that a mechanism for physicians to be involved in EHR design and training, an emphasis the benefits of the EHR, support available from colleagues and clear communication about upgrades would enhance their ability to adapt to the EHR.

## Data Availability

The data analyzed in this study is not included in a repository because it contains interview transcripts.

## Abbreviations

AMC: Academic Medical Center
EHR: Electronic health record
TAM: Technology Acceptance Model
TAM2: Extended Technology Acceptance Model
